# Protocol to generate an allo-transplantable tumor in mouse models using the YUMM1.7 cell line

**DOI:** 10.1016/j.xpro.2026.104449

**Published:** 2026-03-20

**Authors:** Ahmed Rokan, Somaya NoorSaeed, Ariberto Fassati

**Affiliations:** 1 Department of Medical Laboratory, College of Applied Medical Sciences, Prince Sattam Bin Abdulaziz University, Alkharj 11942, Saudi Arabia; 2 Department of Medical Laboratory Sciences, Faculty of Applied Medical Sciences, King Abdulaziz University, Jeddah 22254, Saudi Arabia; 3 Division of Infection & Immunity and Institute of Immunity and Transplantation, The Pears Building, University College London, Pond Street, London NW3 2PP, UK

**Keywords:** Cell culture, Cell isolation, Single Cell, Cell separation/fractionation, Flow Cytometry, Cancer, Immunology, Model Organisms

## Abstract

Here, we present a protocol to generate an allo-transplantable tumor in mouse models using the YUMM1.7 cell line. We describe steps for mouse breeding using syngeneic C57BL/6 and allogeneic BALB/c mice, injecting the YUMM1.7 melanoma cell line into mice, and collecting and dissociating the growing tumors into single cells. We then detail the procedures for injecting the cells into mice for the generation of an allo-transplantable tumor by progressive passaging into more mismatched hosts.

For complete details on the use and execution of this protocol, please refer to Rokan et al.^1^

## Highlights

• Instructions on mouse strains and hybrids used to generate the allo-transplantable tumor
• Steps for *in vitro* culturing of YUMM1.7 cells and the derived lines
• Instructions for tumor cell transplantation and tumor dissociation into single cells
• Guidance on the passaging strategy to generate the allo-transplantable tumor

## Abstract (editor highlights)

Publisher’s note: Undertaking any experimental protocol requires adherence to local institutional guidelines for laboratory safety and ethics.

## Graphical abstract

**Figure:**
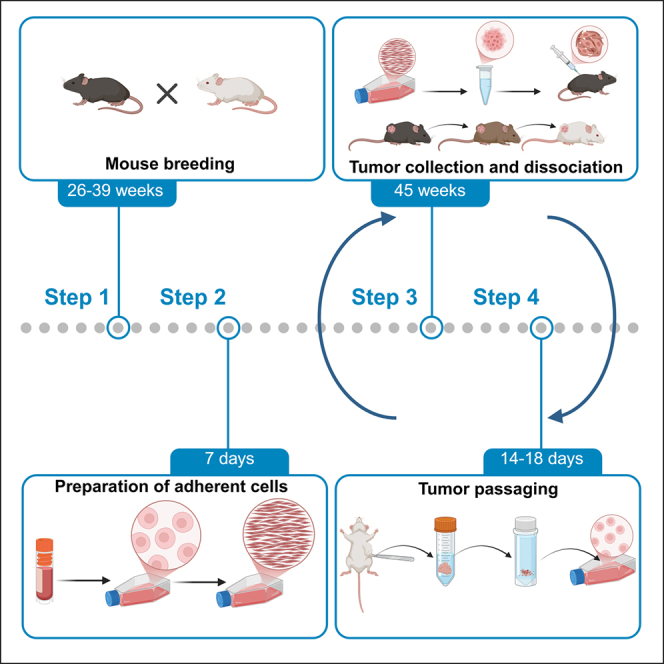

## Before you begin

The histocompatibility barrier is the cornerstone of transplantation immunology. It prevents the transplant of organs between mismatched individuals. MHC-I is extremely polymorphic in humans, further complicating the matter.^2^ However, the discovery of clonally transmissible cancers in dogs, Tasmanian devils, and bivalves that can bypass the histocompatibility barrier challenged this paradigm.^3^ How these tumors evolved this ability is still unknown. Understanding the mechanism of how tumors that are allografts can bypass the histocompatibility barrier could have important implications for the fields of organ transplantation and tumor immunology.

This protocol provides detailed steps for generating allo-transplantable tumors in immunocompetent mice that can bypass the histocompatibility barrier, making it a unique model for studying immune evasion, cancer evolution, and escaping the histocompatibility barrier. This protocol describes the use of C57BL/6 and BALB/c mouse strains for tumor transplantation studies and for breeding of the hybrid strains, as well as CBA/Ca and FVB/N mouse strains to verify the transplantability of evolved tumors. The YUMM1.7 melanoma cell line is used to generate the allo-transplantable tumor due to its well-defined genetic characteristics and relevance to human disease. YUMM1.7 was developed in a male *Pten−/− Cdkn2a−/−*. C57BL/6 mouse with the BRAF^(V600E)^ mutation^4^ that is found in 50% of human melanomas.^4^ We provide step-by-step details on the optimal growth conditions of YUMM1.7 cells and the derived tumors, then their preparation and transport for tumor transplantation in mice. Next, we describe the steps of collecting and dissociating tumors to get viable single cells that can be used for tumor passaging into the next mouse hybrid or strain. Finally, we provide the tumor passaging strategy to evolve a tumor that bypasses the histocompatibility barrier and can grow in mismatched mouse strains.

### Innovation

A study from the 1920s reported the emergence of a tumor that can grow in all mouse strains after being introduced into F1 and F2 hosts.^5^ Studies from the 1950s found that passaging parental cancers in F1 hybrid mice increased the incidence of tumor take in resistant backcross mice by three-fold.^6^^,^^7^ Building on these observations, we designed a tumor passaging strategy in the syngeneic hosts (C57BL/6), F1 mouse (C57BL/6 × BALB/c), F2 mouse (F1 × F1), N2 mouse (F2 × BALB/c) for three sequential rounds, the allogeneic host BALB/c for two sequential rounds, then back to the original host C57BL/6 and two different mouse strains CBA/Ca and FVB/N. This passaging strategy allowed the tumor to gain the ability to escape the histocompatibility barrier and grow in mismatched hosts. Moreover, the protocol utilizes advanced genotyping technologies to ensure mice hybrids are indeed increasing in mismatch.

### Institutional permissions

All the experiments in this paper were performed in compliance with UK Home Office regulations, under project licenses number PP2330953 and PC79FA7AB. The studies were approved and supervised by the BSU at UCL in accordance with the 3R (Replacement, Reduction, Refinement) Principles.

Obtain the required permissions for animal experimentation from the relevant institutions in your country.

### Preparation of mouse models

**Timing: 26**–**39 weeks**

This step describes the mouse strains required in this experiment.

**Figure 1:**
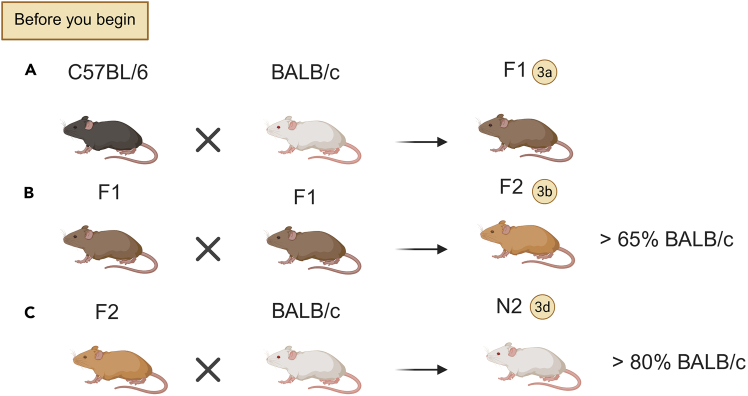
Mouse breeding of F1, F2, and N2 mouse hybrids (A) Schematic depiction for the breeding of F1 mouse hybrid. (B) Schematic depiction for the breeding of F2 mouse hybrid. (C) Schematic depiction for the breeding of N2 mouse hybrid.

1. Acquire 6–8 weeks old mice from the syngeneic mouse strain C57BL/6 (MHC haplotype H-2K^b^ H-2D^b^ H-2L^null^ I-A^b^ I-E^null^), and allogeneic strain BALB/c (haplotype H-2K^d^ H-2D^d^ H-2L^d^ I-A^d^ I-E^d^), for use in tumor transplantation experiments, and 10-14 weeks old mice for breeding of mouse hybrid strains.
2. Acquire 6–8 weeks old CBA/Ca (MHC haplotype H-2K^k^ H-2D^k^ H-2L^null^ I-A^k^ I-E^k^), and FVB/N mouse strains and (MHC haplotype H-2K^q^ H-2D^q^ H-2L^q^ I-A^q^ I-E^null^) to test universal allo-transplantability of evolved tumors.
3. Establish breeding colonies as follows:
  a. To generate the F1 mouse hybrid: acquire 10–14 weeks old (adult) mice from the inbred C57BL/6 mouse strain and the inbred BALB/c mouse strain. Start breeding colony using a monogamous pair or trio, depending on the number of F1 mice desired for the experiments (Figure 1A).
  b. To generate the F2 mouse hybrid (F1 x F1): use 10–14 weeks (adult) male and female F1 hybrids for the breeding colonies (Figure 1B).
  c. Once the F2 mice hybrids are produced, genotype the mice using the Mini Mouse Universal Genotyping Array (MUGA) platform.^8^
    **CRITICAL:** Select F2 mice possessing >65% alleles of the allogeneic strain to increase the mismatch barrier for tumor transplantation experiments.
  d. To generate the N2 mouse hybrid: mate 10–14 weeks (adult) male and female backcross F2 mice with the allogeneic BALB/c mouse strain (Figure 1C).
  e. After the acquisition of the N2 mouse hybrids, genotype the mice with the Mini MUGA platform.
    **CRITICAL:** Select N2 mice with >80% of their alleles coming from the allogeneic strain for tumor transplantation experiments.
    **CRITICAL:** The N2 mouse hybrid will be used in three rounds of transplantation to generate the N2.1, N2.2, and N2.3 tumors. Additionally, the N2 mouse hybrid is highly resistant to the N2.1 and N2.2 tumors; therefore, breed a higher number of the N2 mice for the experiments.

## Key resources table

**Table:** 

REAGENT or RESOURCE	SOURCE	IDENTIFIER
**Antibodies**		

CD45 (1:100 dilution ratio)	Biolegend	103128

**Chemicals, peptides, and recombinant proteins**		

Collagenase, Type IV, powder	Sigma-Aldrich	C4-28-100MG
Trypsin EDTA 0,25%	Corning	25-053-CI
DNase I	Roche	10104159001
DMEM/F-12, HEPES	Gibco	11330032
MEM (Minimum Essential Medium) NEAA (Non-Essential Amino Acids) 100X	Gibco	11140–050
Penicillin/streptomycin (P/S) (100x) (10,000 U/mL/10,000 μg/mL)	Thermo Fisher Scientific	15140122
Fetal Bovine Serum, South American Origin 500mL	Labtech	FCS-SA/500
Dulbecco’s Phosphate-Buffered Saline (DPBS)	Gibco	14-190-144
Dimethyl sulphoxide (DMSO)	ATCC	50-238-3363
Isoflurane	Zoetis	115095
Ethanol	Supelco	46139
Trypan blue solution	Sigma-Aldrich	T8154

**Critical commercial assays**		

MojoSort Mouse CD45 Nanobeads	Biolegend	480028

**Experimental models: Cell lines**		

YUMM1.7 cells	ATCC	CRL-3362
YUMM1.7 passages (F0, F1, F2, N2.1, N2.2, N2.3, Balb-c-1, Balb/c-2, CBA, FVB/N)	This study	N/A

**Experimental models: Organisms/strains**		

6–8 weeks old inbred male and female Mouse/C57BL/6	Charles River and Envigo	N/A
10–14 weeks old inbred male and female Mouse/C57BL/6	Charles River and Envigo	N/A
6–8 weeks old inbred male and female Mouse/BALB/c	Charles River and Envigo	N/A
10–14 weeks old inbred male and female Mouse/BALB/c	Charles River and Envigo	N/A
6–8 weeks old inbred male and female Mouse/CBA/Ca	Envigo	N/A
6–8 weeks old inbred male and female Mouse/FVB/N	Envigo	N/A

**Others**		

Cell Strainers, 40 μm	Corning	431750
50 mL sterile Falcon centrifuge tube	Corning	352070
15 mL sterile Falcon centrifuge tube	Corning	CLS352196
1.5 mL Eppendorf tubes	Invitrogen	AM12400
7 ml Bijou container	WHEATON	216–0980
6-well Clear TC-treated Multiple Well Plates, Individually Wrapped, Sterile	Corning	3516
12-well Clear TC-treated Multiple Well Plates, Individually Wrapped, Sterile	Corning	3513
5 mL Stripette Serological Pipets, Polystyrene, Individually Plastic Wrapped, Sterile	Corning	4051
10 mL Stripette Serological Pipets, Polystyrene, Individually Plastic Wrapped, Sterile	Corning	4101
25 mL Stripette Serological Pipets, Polystyrene, Individually Plastic Wrapped, Sterile	Corning	4251
Mr. Frosty Freezing Container	Thermo Scientific	5100–0001
CRYO.S, 2 ML	GREINER BIO-ONE LTD	122263
CO2 incubator	Binder	CB170
Cell culture flask, T-75, surface: Cell+, filter cap	STARLAB (UK) LTD	83.3911.502
Cell culture flask, T-25, surface: Cell+, filter cap	STARLAB (UK) LTD	83.3910.302
Stainless Steel. Bochem Dissecting Set includes eight pieces.	FISHER SCIENTIFIC	10430733
BD Microlance stainless steel needles 26G	BD	10753785
Plastipak disposable syringe 1mL concentric sterile	BD	SYR6000
Bochem 18/10 stainless steel forceps, blunt tip	FISHER SCIENTIFIC	10663341
Millicell disposable hemocytometer	Millipore	MDH-2N1
Digital caliper	Fisherbrand	11783217
MiniARCO rodent shaver	WAHL	CL8787-KIT
Ear punch 2mm	Wpiinc	500075
Accuris mini balance for mice	Accuris	SCL-W1005-100
Inverted microscope	Leica Microsystems	DMi1
CliniScav+ Anaesthesia benchtop system	Clinipath	N/A
Clinipath CO2 chambers	Clinipath	N/A
CASY cell counter	OMNI Life Science	N/A
Standard LA precision balance	METTLER TOLEDO	01-804-204
VWR VWB2 water baths	VWR	462–0556
Class II biological safety cabinet	NUAIRE	NU-560
Animal transfer station	Allentown	ATS5
Microcentrifuge 5425 R	Eppendorf	5406000364
Benchtop centrifuge 5910 Ri	Eppendorf	5943000061
BD Fortessa flow cytometers.	BD	N/A

**Software and algorithms**		

Biorender	Biorender	N/A
FlowJo-v10	FlowJo LLC	N/A

## Materials and equipment

**Table:** Complete media

Reagent	Final concentration	Amount
DMEM/F-12, HEPES	88%	440 mL
FBS	10%	50 mL
P/S	1%	5 mL
MEM NEAA	1%	5 mL
Total	N/A	500 mL

Store at 4°C for up to 1 month.

**Table:** Cryopreservation media

Reagent	Final concentration	Amount
Complete media	90%	45mL
DMSO	10%	5 mL
Total	N/A	50 mL

Store at 4°C for up to 1 month.

**Table:** Transport media

Reagent	Final concentration	Amount
1x DPBS	98%	49mL
FBS	1%	0.5 mL
P/S	1%	0.5 mL
Total	N/A	50 mL

Store at 4°C for up to 1 month.

**Table:** Collagenase type IV dissociation buffer

Reagent	Final concentration	Amount
1x DPBS	N/A	9.9 mL
Collagenase	200 U	1 G
P/S	1%	0.1 mL
Total	N/A	10 mL

Store at 4°C for up to 1 month.

**Table:** DNAse I dissociation buffer

Reagent	Final concentration	Amount
1x DPBS	N/A	100 mL
DNAse I	1 mg/mL	100 mg
Total	N/A	100 mL

Store at 4°C for up to 1 month.

**Table:** 0.05% Trypsin dissociation buffer

Reagent	Final concentration	Amount
0.25% Trypsin	0.05%	2 mL
1x DPBS	N/A	8 mL
Total	N/A	10 mL

Store at 4°C for up to 1 month.

## Step-by-step method details

### Preparation of YUMM1.7 cells for injection

**Timing: 7 days**

This step enables you to prepare tumor cells for transplantation in mice. It’s critical that tumor cells are alive and growing in the log phase.

1. Acquire the YUMM1.7 cell line from the ATCC.
2. Thaw the cells in 37°C and grow them in 25 cc flasks using DMEM/F12 + 10% FBS + 1% MEM NEAA + 1% P/S complete media.
3. Perform all quality checks required to verify the cell line is contamination-free and of the required background.
4. Expand the cell line in culture in 75 cc flasks using DMEM/F12 complete media until the cells are 75%–90% confluent to make sure the cells are still in the log growth phase.

***Note:*** It takes YUMM1.7 cells 48–72 h to reach confluency depending on the starting seeding density of the cells.

***Note:*** Grow 2x the number of cells required for mouse transplantation experiments to prepare for unexpected loss of cells (i.e., if your experiment includes 10 mice and the number of cells to be injected for each mouse is 1 million cells, prepare 20 million cells instead of 10 million cells).

5. Remove the culture media using a serological pipette.
6. Wash the cells 2× by using 1× DPBS gently to prevent disrupting the cells.
7. Detach the cells using an equal amount of 1× DPBS and 0.25% trypsin-EDTA to cover the cells.
8. Incubate the cells in a 37°C and 5% CO2 incubator for 5 min.
9. Check the cells under an inverted microscope to confirm that the cells are fully detached from the surface of the flask.
10. Stop the trypsin activity by adding 4× the amount of trypsin from the complete media used to grow the cells.
11. Collect the cells from the different flasks into a sterile 50 mL Falcon tube.
12. Centrifugate the cells at 350 × *g* for 5 min at 25°C.
13. Remove the supernatant by aspiration with a serological pipette.
14. Wash the cells with 1x DPBS.
15. Centrifugate the cells at 350 × *g* for 5 min at 25°C.
16. Remove the supernatant by aspiration with a serological pipette.
17. Resuspend the cells in an appropriate amount of 1× DPBS.
18. Count the cells by using a hemocytometer or automated counters such CASY cell counter.

**CRITICAL:** Check cell viability; it’s essential that cell viability exceeds 90% for optimal tumor cell engraftment.

19. Resuspend the cells in sterile 1× DPBS at 1 million cells/100 μl in sterile 1.5 mL Eppendorf tubes.

***Note:*** Some protocols recommend the use of Matrigel with PBS at 1:1 ratio for tumor transplantation experiments. As Matrigel contains mouse derived growth factor and can artificially enhance tumor take rates or accelerate tumor growth,^9^^,^^10^ we don’t recommend the use of Matrigel in this protocol and adhere to using DPBS suspended tumor cells for injection to allow for the natural evolution of tumors that can escape the allogeneic rejection.

**Figure 2:**
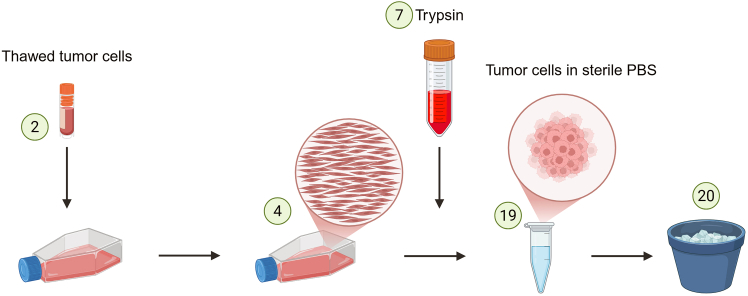
Cell culturing of YUMM1.7 cell line

20. Transfer the resuspended tumor cells to ice to preserve the cells from dying during transport (Figure 2).

### YUMM1.7 cell transplantation into C57BL/6 mouse

**Timing: 14**–**18 days**

This step enables you to transplant live tumor cells in mice without the development of infections, ulcers, or other adverse side effects that could affect the mouse’s health and welfare.

**CRITICAL:** All work must be done in a class II Biological Safety Cabinet (BSC) in a sterile condition to prevent the contamination of the cells or the development of infections in mice.

**CRITICAL:** Equal ratios of 6–8 weeks old male and female mice should be used in all tumor transplantation experiments to account for any potential sex-specific effect in the allograft immune response.

21. Take out the YUMM1.7 cells from ice and allow them to warm up to 25°C for injections.
22. Once tumor cells reach 25°C, mix the cells gently, load up your syringes with the appropriate amount for injection, and eliminate air bubbles before injection.

***Note:*** Use a smaller needle gauge for subcutaneous injections into mice, 25–27 G.

***Note:*** Use needle length between ⅜–½ inch (9–13 mm) for the injection.

23. Check the health of all mice before starting the experiment, and make sure there are no signs of illness or distress.
24. Shave the left flank of the mouse using a rodent shaver for better visibility of the site of the injection and to better track tumor growth.

***Optional:*** Mice could be shaved ahead of time before injection.

***Note:*** If you are working alone, it’s easier to shave the mouse under anesthesia using an isoflurane vapor system.

25. Record the weight of the mouse prior to tumor injection and use it as a record to follow up on the mouse’s health.

***Note:*** If a mouse loses 20% or more of its weight during the experiment, you must euthanize the mouse and end the experiment on that mouse.

26. Inject 1 million tumor cells subcutaneously into the shaved area of C57BL/6 mouse.

**CRITICAL:** Make sure the injection is not too deep to prevent injecting the cells into the muscle tissue, nor too superficial in the intradermal layer.

***Note:*** If using multiple tumor cell samples, mark the mice by using ear clip marking or other methods.

***Note:*** If you are injecting mice under anesthesia, move the mouse into the recovery area and observe it until it regains consciousness.

27. Record all the details of the experiment, such as the weight of mice before injection, the tumor cell type and volume each mouse received, the dates of the experiment in your lab notebook, and the mouse cage card.
28. Check on all the mice in the experiment for signs of illness or distress at the end of the day of the experiment and the next day.
29. Check the mice for general health and tumor growth on a daily or bidaily basis and measure and record the weights of the animals.

***Note:*** If a mouse develops diarrhea and dehydration, loss of muscles, or apparent skeletal structure after tumor injection, euthanize the mouse and end the experiments.

30. If tumor growth is noticed, measure the tumor size by using a caliper and record it.

**CRITICAL:** Check with the regulating authorities in your country for the maximum tumor size and diameter allowed before the humane endpoint of the experiment must be met.

**Figure 3:**
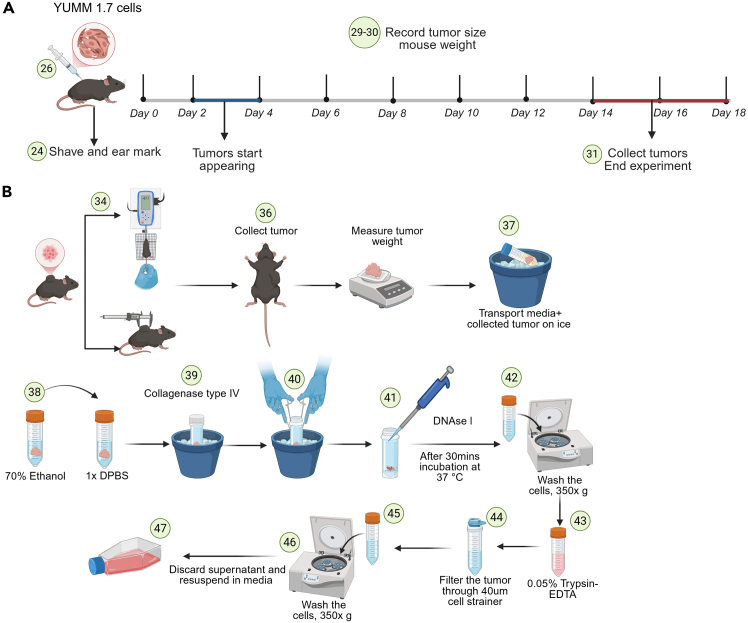
Production of F0 tumor line in C57BL/6 mouse (A) Schematic diagram for the procedure of tumor transplantation experiment. (B) Workflow for tumor collection and dissociation into single cells.

31. Allow the tumors to grow for 14–18 days before ending the experiment and collecting tumors (Figure 3A).

### Tumor collection and dissociation into viable single cells

**Timing: 2**–**5 h**

This step allows you to collect and preserve tumor cells to prevent cell death during transportation. Then, dissociate tumors to get viable tumor single cells that can grow *in vitro* in cell culture for further analysis and tumor passaging experimentation.

32. After tumor growth for 14–18 days after injection, tumors are ready for collection.
33. Prepare tumor transport media containing 1x DPBS and 1% FBS and 1%P/S.

***Note:*** The addition of 1% FBS enhances the viability of tumors, while 1% P/S is used to prevent bacterial growth in the tumor tissue.

34. Measure the tumor size and weight of the mouse and record them.
35. Euthanize mice by CO2 overload and cervical dislocation.
36. Dissect the mouse using sterile surgical tools, collect the tumors and measure their weights.

**CRITICAL:** Make sure you only collect the tumor without the surrounding tissue to prevent the contamination of tumor cells with other non-related cells.

37. Place the collected tumors in the transport media and place them on ice.

***Note:*** Tumors should be fully submerged in the transport media.

38. Working in sterile conditions in a class II BSC, quickly dip the tumors in freshly prepared 70% ethanol, then rinse in sterile 1× DPBS.
39. Transfer the tumor to a Bijou tube on ice and add 1mL of 1× DPBS containing 200U of collagenase IV + 1% Pen/strep.
40. Mince the tumor on ice with two small scissors into small pieces (1mm or less).
41. Add 1 mL of 1 mg/mL DNase I to the sample and mix, incubate at 37°C for 30 min.
42. Wash the cells with cold sterile 1x DPBS and centrifuge at 350 × *g* at 25°C.
43. Digest with 1mL of 0.05% Trypsin-EDTA, incubate for 5 min at 37°C.
44. Filter the digested tumors through a 40μm cell strainer to obtain single cells suspension.^11^

***Note:*** To help cells go through the strainer, add cold sterile 1x DPBS slowly.

45. Dilute cells with 30 mL of cold sterile 1× DPBS.
46. Centrifugate the cells at 350 × g, then discard the supernatant.
47. Resuspend the cells in complete DMEM/F12 media and culture the cells in 25 cc flasks at 37°C and 5% CO2 incubator.
48. Monitor the cells for cell attachment and growth. Upon the attachment of a sufficient number of cells, replace the media to eliminate debris and immune cells existing in the dissociated tumor.

***Note:*** Alternatively, immune cells can be eliminated by magnetic sorting with antibodies against pan leukocyte marker CD45 after obtaining the single-cell suspension.

49. The tumor produced by YUMM1.7 growth in a C57BL/6 mouse is called F0, and it is used for passaging into the F1 mouse hybrid (Figure 3B).

### Generation of an allo-transplantable tumor by progressive passaging

**Timing: 45 weeks**

This step allows you to generate a tumor that can grow in mismatched hosts. This is achieved by transplanting the tumor in mice with an increasingly greater mismatch.

**Figure 4:**
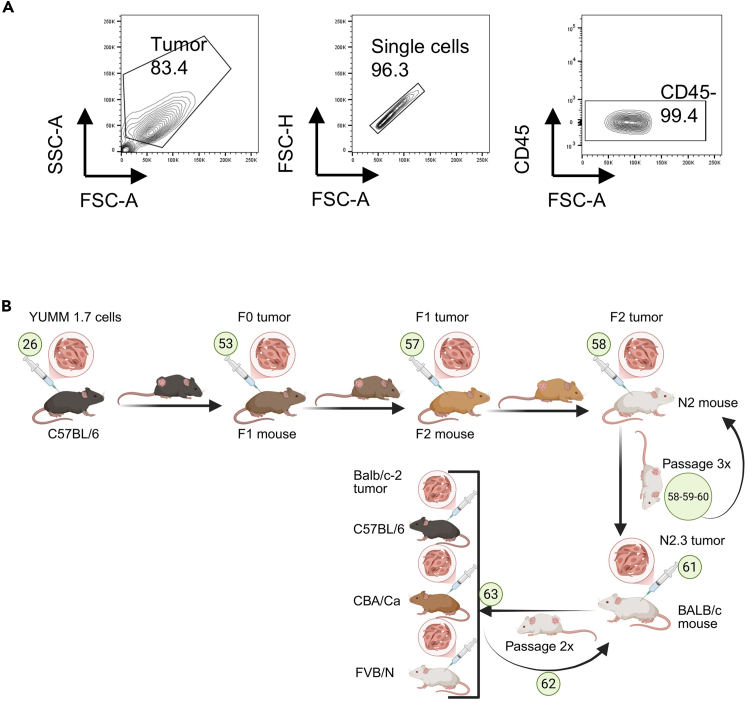
Tumor passaging strategy to generate allo-transplantable tumor (A) Flow cytometry plots showing the gating strategy for tumors cells before tumor injection experiments. (B) Schematic depiction of the tumor passaging strategy for generation of allo-transplantable tumor.

50. Verify that tumor cells are free from immune cells by using flow cytometry staining against anti-CD45 antibodies (Figure 4A).
51. Expand the F0 tumor cells in complete DMEM/F12 media until they reach the required number for injection into the F1 mouse.
52. Prepare the cells for injection as described in steps 5–20.
53. Inject 6–8 weeks old F1 mice with 1 million cells of F0 tumor cells into the shaved flank area.
54. Monitor tumor growth as described in steps 30–32.
55. Collect the F1 tumors into tumor transport media.
56. Dissociate the F1 tumors as described in steps 38–47.
57. Proceed to inject the F1 tumor cells into the F2 mouse to generate the F2 tumors following the same steps.

***Note:*** No significant rejection of the F0, F1, and F2 tumors is noticed; therefore, use the minimal number of mice in these experiments to achieve statistical significance.

58. Generate the N2.1 tumor by passaging the F2 tumor cells in the N2 mouse hybrid.
59. Generate the N2.2 tumor by passaging the N2.1 tumor cells in the N2 mouse hybrid.

***Note:*** The percentage of N2.1 and N2.2 that grow without rejection in the N2 mouse hybrid is 40% and 46%, respectively.^1^ Decide the number of mice used for these experiments based on these percentages and adhering to the 3R principles.

60. N2.3 tumors are generated by passaging the N2.2 tumor cells in the N2 mouse hybrid.

**CRITICAL:** If the tumor take is low in a mouse strain, inject the tumors that grew in this mouse strain into the same type of mouse to allow better tumor adaptation to this type of mouse, therefore increasing the tumor take ratio.

61. Proceed to inject the N2.3 tumor cells into BALB/c mouse to get the Balb/c-1 tumor passage.
62. Inject the Balb/c-1 tumor cells into BALB/c mouse to generate Balb/c-2 tumors.

***Note:*** Tumor growth in BALB/c mouse indicates allo-transplantability.

63. To confirm the growth of evolved tumors in different mouse strains, transplant Balb/c-2 tumor cells into CBA/Ca, FVB/N mice, and the syngeneic strain C57BL/6 (Figure 4B).

## Expected outcomes

During the early tumor passages F0, F1, and F2, no significant rejection is expected and ≥90% of tumors should grow. Once F2 tumors are passaged into N2 hybrid mice, which have 80–90% of the genome coming from the allogeneic background, rejection rates increase, as only around 40% of tumors are expected to grow. Repeated passaging of tumors that grew in the N2 mouse hybrid strain should increase the tumor take rates. When the percentage of tumors that can grow at N2 hybrids without rejection reached 80–90%, these tumors should be able to grow in the allogeneic hosts (BALB/c) at high rates ≥ 75%. Repeated passaging in the allogeneic hosts should also increase the take rates. Once a tumor can grow in the allogeneic host at rates exceeding 90%, it can grow in other mouse strains that are mismatched to both the syngeneic and allogeneic mouse strains used.^1^

Following tumor dissociation, tumor cells will take between 1–7 days to start attaching to the surface of the flask, and cell replication might start slowly, then accelerate in the following days. YUMM1.7 cells and their derived tumor cells exhibit an epithelial-like morphology after attachment. After 7 days of attachment and replication, the cells should start growing at a normal rate, where they double every 12 h.

## Limitations

A potential limitation of this passaging strategy protocol is that it might not be applicable universally to all types of tumors. This study was done on the melanoma cell line YUMM1.7. Studies from the 1950s used a mouse mammary carcinoma that showed successful adaptation to backcross mice.^6^ However, when the undifferentiated spindle-cell carcinoma was passaged into F1 hybrids, then to backcrossed mice, it didn’t show similar adaptation capabilities.^12^ Additionally, this protocol is designed to evolve tumors that escape acute allogeneic rejection, but it is unknown whether it can lead to escaping chronic allogeneic rejection.

## Troubleshooting

### Problem 1

Low tumor engraftment rates when higher rates are expected (step 21–31).

### Potential solution

Low tumor cell engraftment rates could be caused by multiple reasons, such as the use of tumor cells with a high percentage of dead cells, tumor cells passaged for too long in culture, or contamination of tumor cells with immune cells when dissociated tumors are used. Before tumor injection, verify that ≥ 90% of cells are viable by using trypan blue staining or another method. Additionally, use cells with a low passage number <10 to avoid phenotypic changes that happen to cells when passaged *in vitro*. Finally, when dissociated tumors are used early after dissociation, stain the cells against CD45 to confirm the lack of immune cells before injection. If immune cells exist, allow the tumor cells to grow in culture for a few more passages until the immune cells are cleared, or eliminate the immune cells by using MACS sorting against CD45.

### Problem 2

Ulceration at the tumor subcutaneous injection site (step 21–31).

### Potential solution

The development of ulceration at the injection site could be due to technical or biological reasons. The injection of the tumor too superficially at the intradermal instead of the subcutaneous layer, the use of high volume or high cell number, and the injection with a high percentage of dead cells and/or debris are the main technical causes of the development of ulceration at the injection site. To minimize the risk of ulceration development, inject the tumor cells into the subcutaneous layer. Use volumes ≤100 μl and optimize the cell number depending on the tumor type used. Use cells that are ≥ 90% viable, and when freshly dissociated tumors are used, wash the tumor cells carefully until cellular debris from the dissociation process is fully removed.

### Problem 3

Dissociated tumor sample exhibiting slower replication in culture for a prolonged period (step 32–49).

### Potential solution

Failure of a tumor cell sample to exhibit similar replication kinetics to other tumors from the same passage could be due to contamination of the tumor sample with other cells, such as muscle cells, during dissection. Perform Short Tandem Repeat genotyping (STR) profiling to check the tumor sample genetic background compared to other tumor samples.

### Problem 4

Dissociated tumor cells are dead and not recoverable after freezing (step 32–49).

### Potential solution

This is likely caused by poor freezing procedure or freezing the cells immediately after dissociation. It is recommended to use controlled-rate cryopreservation (−1°C/min) by using an isopropanol freezing container. Tumor cells should be frozen in freezing medium containing 10% DMSO. Freezing tumor cells immediately after dissociation will likely result in high cell loss, which might lead to cells not recovering from freezing. To solve this problem, allow the tumor cells to grow in culture for a few more days or passages, and freeze the tumor cells when they are around 90% confluent.

### Problem 5

Low rejection rate when passaging F2 tumors in N2 mouse (step 58).

### Potential solution

This is likely due to using N2 mice that are not >80% of the BALB/c background. Not increasing the mismatch could cause tumors to not be able to grow in BALB/c mice in later steps. To solve this problem, check the genetic background by using genotyping services such as Mini MUGA and use N2 mice that have a proportion of BALB/c >80%.

## Resource availability

### Lead contact

Further information and requests for resources and reagents should be directed to and will be fulfilled by the lead contact, Ahmed Rokan (a.rokan@psau.edu.sa).

### Technical contact

Technical questions on executing this protocol should be directed to and will be answered by the technical contact, Ahmed Rokan (a.rokan@psau.edu.sa).

### Materials availability

The evolved tumor cell line produced by this study, Balb/c-2, is in the process of being deposited to ATCC. For further requests regarding material availability, contact Ahmed Rokan (a.rokan@psau.edu.sa).

### Data and code availability

This study did not generate large datasets or code.

## Acknowledgments

The authors extend their appreciation to 10.13039/100009392Prince Sattam bin Abdulaziz University for funding this research work through project no. 10.13039/100009392PSAU/2025/01/36583. The graphical abstract and figures were created using Biorender.com.

## Author contributions

A.F. and A.R. designed and conceptualized the tumor passaging strategy. A.R., A.F., and S.N. performed and optimized the tissue culturing experiments. A.R., A.F., and S.N. performed and optimized the tumor dissociation protocol. A.R. performed and optimized the tumor transplantation experiments. A.R. and S.N. prepared the figures of this manuscript. A.R. wrote the original draft. A.R., A.F., and S.N. reviewed and edited the manuscript.

## Declaration of interests

The authors declare no competing interests.

## Declaration of generative AI and AI-assisted technologies in the writing process

During the preparation of this work, the authors used ChatGPT-5.2 to refine the manuscript. After using this tool, the authors reviewed and edited the content as needed and take full responsibility for the content of the published article.
